# GC-MS-Based Volatile Metabolomics and Quantitative Trait Locus Mapping of Aroma Components in ‘Muscat Hamburg’ Grape

**DOI:** 10.3390/plants15172708

**Published:** 2026-09-03

**Authors:** Minmin Li, Peng-Tao Shi, Nan Jia, Yong-Gang Yin, Yan Sun, Bin Han, Chang-Jiang Liu, Shu-Li Han, Ying-Jie Wang, Xin-Yu Wang, Jian-Rong Feng

**Affiliations:** 1 Xinjiang Production and Construction Corps Key Laboratory of Special Fruits and Vegetables Cultivation Physiology and Germplasm Resources Utilization, College of Agriculture, Shihezi University, Shihezi 832000, China; 15903395217@163.com; 2 Changli Institute of Pomology, Hebei Academy of Agriculture and Forestry Sciences, Changli, Qinhuangdao 066600, China; 2018051164@nwafu.edu.cn (P.-T.S.); nannanjia123@163.com (N.J.); yinyonggang@126.com (Y.-G.Y.); sycoolhi@163.com (Y.S.); hanbin986@163.com (B.H.); liuchj2008@163.com (C.-J.L.); h15633401356@163.com (S.-L.H.); 17733503931@163.com (Y.-J.W.)

**Keywords:** Muscat Hamburg, SLAF-seq, aroma, *VvDXS*, *VvGGPPS*

## Abstract

The muscat aroma of grape is largely determined by terpenoid volatiles. However, its genetic basis in ‘Muscat Hamburg’ remains unclear. We performed comparative volatile metabolomics between ‘Muscat Hamburg’ and ‘Red Globe’ across berry development, identifying 127 differentially abundant metabolites, with upregulated ones enriched in the monoterpenoid pathway. Using an F1 segregating population (264 progeny), we quantified four key monoterpenoids (α-terpineol, linalool, geraniol, and *cis*-linalool oxide) over two consecutive years. Using a high-density genetic map with 7872 SNP markers generated via SLAF-seq, we detected stable major QTLs for α-terpineol, linalool, and geraniol on LG5, and a novel QTL for *cis*-linalool oxide on LG14. Expression analysis revealed that the transcript levels of *VvDXS* and *VvGGPPS1* were significantly higher in ‘Muscat Hamburg’ than in ‘Red Globe’ at ripening. Heterologous overexpression in tomato enhanced terpenoid aroma accumulation without affecting fruit development. These results indicate that *VvDXS* and *VvGGPPS1* play a crucial role in the formation of the aroma in the ‘Muscat Hamburg’ variety.

## 1. Introduction

Grape (*Vitis vinifera* L.) is a globally important horticultural crop, and fruit aroma is a core determinant of berry quality, commodity value, and consumer preference [1,2]. Grape aroma is a complex trait shaped by various compounds, including terpenes, volatile aliphatic compounds, aromatic compounds, pyrazines, and sulfur-containing compounds [3,4]. Based on aromatic profiles, grape varieties are generally classified into three types: muscat varieties (intensely flavored muscats), strawberry varieties (non-muscat but aromatic varieties), and neutral varieties (not dependent upon monoterpenes for their flavor) [5,6]. Among these, terpenes and aldehydes dominate in the skin of strawberry and muscat cultivars, whereas esters and aldehydes are the main volatiles in the skin of neutral cultivars [7]. And methyl anthranilate, a typical non-terpenoid aromatic ester responsible for the “foxy” flavor, is a characteristic aroma compound of non-V. vinifera grapes, such as American varieties and their hybrids [8]. Muscat grapes are characterized by their typical rose-like aroma, which primarily originates from low-molecular-weight compounds, including sesquiterpenes and, in particular, monoterpenes. These monoterpenes are abundant in the Eurasian grapevine *Vitis vinifera* [6,9]. However, the precise regulatory mechanism underlying monoterpene biosynthesis and accumulation in ‘Muscat Hamburg’ grapes remain largely unclear.

Terpenoids constitute a large family of compounds built from isoprene (C5) units [10,11]. In grapevines, terpenoids are synthesized via two distinct pathways: the cytosolic mevalonate (MVA) pathway and the plastidial 2–*C*–methyl–d–erythritol–4–phosphate (MEP) pathway. Monoterpenes and diterpenes are mainly produced through the MEP pathway, whereas sesquiterpenes are predominantly synthesized in the cytoplasm via the MVA pathway [10]. In the early stage of the MEP biosynthesis pathway, 1-deoxy-D-xylulose-5-phosphate synthase (DXS) is recognized as a key rate-limiting enzyme; in the middle stage, geranyl diphosphate synthase catalyzes the condensation of isopentenyl pyrophosphate and its isomer dimethylallyl pyrophosphate to form geranyl pyrophosphate (GPP) [11,12,13]. GPP then serves as a substrate for terpene synthases to generate monoterpenes [12]. GPP plus the subsequent addition of isopentenyl pyrophosphate molecules gives first C15—farnesyl pyrophosphate; and then C20—geranylgeranyl pyrophosphate (GGPP) [14].

Among all terpenoids, only low-molecular-weight monoterpenes—such as linalool, nerol, geraniol, citronellol, and limonene—significantly influence the flavor of grape berries and wine, whereas larger diterpenes and triterpenes lack aromatic properties [15]. By measuring the concentrations of linalool, nerol, and geraniol—three monoterpenes strongly correlated with the rose-scented aroma—quantitative trait locus (QTL) scanning of five hybrid populations revealed QTLs for these three compounds on linkage groups 1, 2, 5, 7, 12, 13, and 16 [16]. Subsequent studies using high-resolution gas chromatography–mass spectrometry (HRGC–MS) on two F1 progenies over three and two years identified a major QTL co-localizing on LG5 that controls the accumulation of all three monoterpenes; this QTL encodes DXS [17]. In another study conducted in the same year, the QTL mapping of terpene compounds in two descendant populations derived from self-pollination of ‘Muscat Ottonel’ and ‘Gewurztraminer’ indicated that the major-effect gene controlling the concentration of monoterpenes is *DXS* on LG5 [18]. Further investigation showed that a single nucleotide substitution (G→T) at position 1822 in the coding region of *VvDXS* on chromosome 5 affects enzyme activity and determines high or low monoterpene content [19]. However, whether this LG5 QTL interval contributes to muscat aroma formation in ‘Muscat Hamburg’ remains unknown.

Geranylgeranyl diphosphate synthase (GGPPS) catalyzes the formation of GGPP, a central precursor for chlorophylls, carotenoids, and gibberellins [20,21,22]. GGPPS enzymes can be classified into three types: type I contains a large aromatic amino acid at the fourth or fifth position before the first aspartate-rich motif (FARM); type II has a two-residue insertion in the FARM but lacks an aromatic residue at the fourth or fifth position before FARM; and type III also lacks an aromatic residue before FARM and has no extra residue insertion in FARM [23,24]. Structurally, GGPPS enzymes predominantly function as homodimers [13,25,26,27,28]. However, heterodimeric and oligomeric forms have also been reported, for example, in rice, *Mentha piperita*, and *Homo sapiens* [29,30,31]. In rice thylakoid membranes, GGPPS1 activity resides in a heterodimeric enzyme consisting of one OsGGPPS1 subunit and GGPPS recruiting protein (OsGRP), which is most similar to members of the small subunit II subfamily of geranyl diphosphate synthase. OsGRP determines the sub-organellar localization of OsGGPPS1 and directs it to large protein complexes on the thylakoid membrane. Genomic and biochemical analyses indicate that OsGRP functions to recruit OsGGPPS1 from the stroma to the thylakoid membrane, thereby providing a mechanism that directs the flux of GGPP towards chlorophyll synthesis [29]. While the role of DXS in muscat aroma has been extensively documented [17,18,22], the involvement of GGPPS in regulating monoterpenoid accumulation in muscat grapes has been described in only a few reports [22].

In this study, to elucidate the metabolic regulation mechanism underlying the characteristic aroma of ‘Muscat Hamburg’, we first performed a comparative volatile metabolomics analysis between the muscat varieties ‘Muscat Hamburg’ and the neutral varieties ‘Red Globe’. Using a GC-MS-based platform and relative database, a total of 442 metabolites were detected. This analysis revealed that 84 metabolites increased significantly during berry ripening in ‘Muscat Hamburg’ compared with that in ‘Red Globe’. Based on literature reports, two monoterpene metabolites (*cis*-linalool oxide and α-terpineol) were selected, along with geraniol and linalool (both are the key monoterpenols responsible for the aroma attributes of grape berries [2,7,17]), as trait indicators for subsequent QTL mapping. We then used an F1 population derived from a cross between the muscat variety ‘Muscat Hamburg’ and the neutral variety ‘Red Globe’ to investigate the genetic basis of aroma in ‘Muscat Hamburg’ grape. Phenotypic data for *cis*-linalool oxide, α-terpineol, linalool, and geraniol contents were collected over two consecutive years (2019 and 2020), using gas chromatography–mass spectrometry (GC-MS). Based on specific-locus amplified fragment sequencing (SLAF-seq), a high-density genetic map was constructed, enabling the identification of quantitative trait loci (QTLs) associated with *cis*-linalool oxide, α-terpineol, and geraniol contents. Candidate genes within major QTL intervals were selected, and their expression dynamics were analyzed by RT-qPCR. The most promising candidates, *VvDXS* and *VvGGPPS1*, were further characterized through overexpression assays. This work provides new insights into the molecular genetic regulation of aroma in ‘Muscat Hamburg’ grape and offers valuable targets for molecular breeding in grapes.

## 2. Results

### 2.1. Comparative Volatile Metabolomic Profiling Between ‘Muscat Hamburg’ and ‘Red Globe’ Grape Berries

To elucidate the metabolic basis of the characteristic aroma difference between the aromatic cultivar ‘Muscat Hamburg’ and the neutral cultivar ‘Red Globe’, we performed a comprehensive volatile metabolomics analysis of grape berries across five consecutive developmental stages using GC-MS. A total of 442 volatile metabolites were detected and identified across all samples, covering terpenoids, aliphatic alcohols/aldehydes, aromatic compounds, esters, and other volatile species associated with grape aroma. Hierarchical clustering analysis (HCA) revealed a clear separation of volatile metabolite profiles between the two cultivars, with developmental stage as the secondary clustering factor (Figure S1). The metabolite abundance patterns of ‘Muscat Hamburg’ at late ripening stages were significantly distinct from those of ‘Red Globe’, indicating a dramatic accumulation of aroma-related metabolites during the ripening process of the muscat cultivar. Compared with ‘Red Globe’, the terpene compounds, esters, and aldehydes related to aroma in ‘Muscat Hamburg’ showed a significant upward trend, suggesting the differences in aroma components between ‘Muscat Hamburg’ and ‘Red Globe’ (Figure S1).

**Figure 1:**
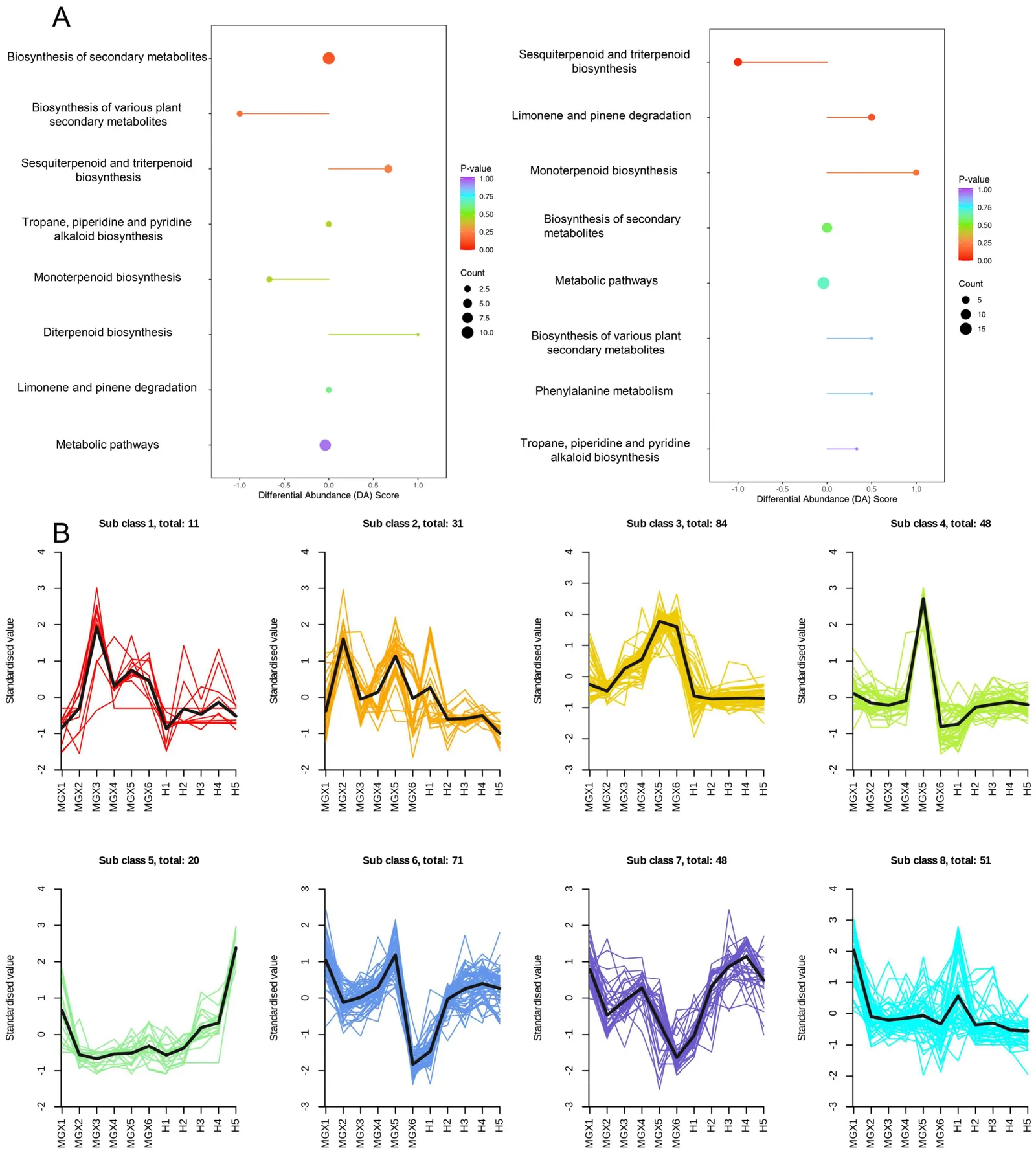
Comparative volatile metabolomics analysis of berry fruits between ‘Muscat Hamburg’ and ‘Red Globe’. (**A**) Differential pathway enrichment analysis based on Differential Abundance Score (DA Score). The vertical and horizontal axes indicate pathway names and DA Score, respectively. Left panel, ‘Red Globe’; Right panel, ‘Muscat Hamburg’. The colors of the line segments and dots indicate the magnitude of the *P*-value. The closer they are to red, the smaller the *P*-value; the closer they are to purple, the larger the *P*-value. (**B**) K-Means clustering analysis of differential volatile metabolites across berry developmental stages. H1-H5 represent five developmental stages of ‘Red Globe’ berries; MGX1-MGX5 represent five developmental stages of ‘Muscat Hamburg’ berries; MGX6 represents the overripening stage of ‘Muscat Hamburg’ berries.

Differential pathway enrichment analysis based on Differential Abundance Score (DA Score) showed that the upregulated metabolites in ‘Muscat Hamburg’ were predominantly enriched in the monoterpenoid biosynthesis pathway (Figure 1A), which is the core metabolic pathways for grape-aroma formation. K-means clustering analysis further classified the DAMs into six clusters based on their abundance-change patterns during berry development (Figure 1B). Cluster 3, which contained 84 metabolites, including the key monoterpenes *cis*-linalool oxide and α-terpineol, showed a continuous upregulation trend during berry ripening in ‘Muscat Hamburg’ but remained at low abundance in ‘Red Globe’, suggesting that these metabolites are the core contributors to the aroma formation. Further quantitative analysis of the core aroma compounds revealed that the average content of α-terpineol in ‘Muscat Hamburg’ was 8.03 times that of ‘Red Globe’, and the difference between the two groups was extremely significant. As the fruit matured, the difference in the content of α-terpineol between ‘Muscat Hamburg’ and ‘Red Globe’ continued to widen, rising from 1.7 times in period 1 to 28.45 times in period 5, showing a clear temporal effect. At the same time, the average content of *cis*-linalool oxide in ‘Muscat Hamburg’ was 7.40 times that of ‘Red Globe’, and the difference reached 21.26 times in period 5, showing a high degree of synergy with the accumulation pattern of α-terpineol. The specific high accumulation of these two terpenoid substances suggests that they are important metabolic bases for the formation of the aroma in ‘Muscat Hamburg’. Combined with literature reports on grape aroma-active compounds [32], we selected *cis*-linalool oxide, α-terpineol, linalool, and geraniol as the core phenotypic indicators for subsequent genetic mapping analysis.

### 2.2. Phenotypic Variation in Key Monoterpenoid Aroma Traits in the F1 Population

To investigate the genetic regulation mechanism of monoterpenoid aroma traits, we constructed an F1 segregating population consisting of 264 progenies derived from a cross between ‘Muscat Hamburg’ and ‘Red Globe’. The concentrations of four key monoterpenoid aroma components (α-terpineol, linalool, geraniol, and *cis*-linalool oxide) in mature berries of the F1 population were quantified using GC-MS over two consecutive growing seasons (2019 and 2020).

**Figure 2:**
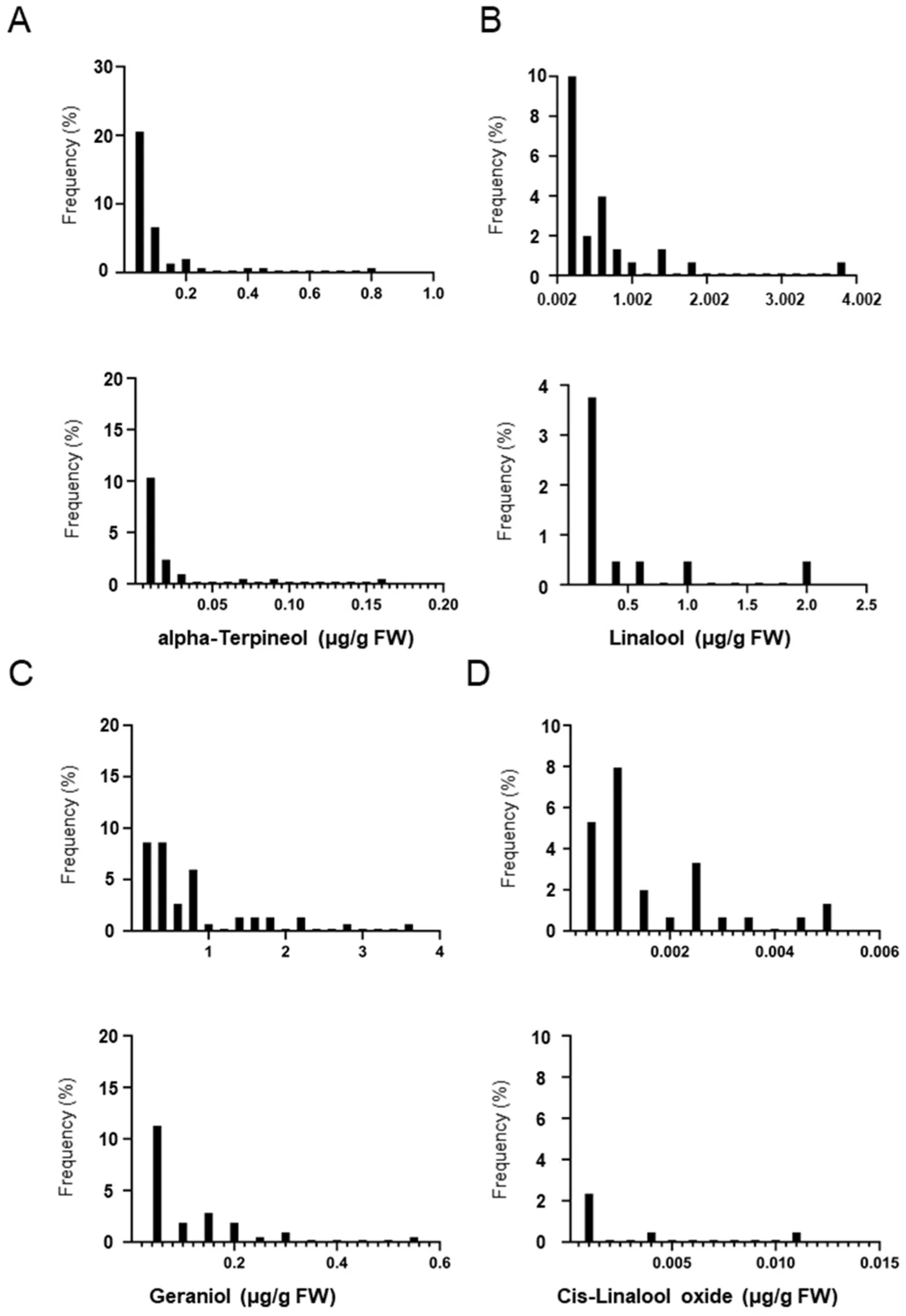
Frequency distribution of key monoterpenoid aroma-component concentrations in the F1 segregating population derived from ‘Muscat Hamburg’ × ‘Red Globe’. Concentrations are presented as μg/g FW of fractions in mature berries, data were collected over two consecutive growing seasons (2019: top panel; 2020: bottom panel), and samples with 0 concentration were excluded from the analysis. The y-axis represents relative frequency (proportion of individuals in the F1 population). Histograms were constructed with compound-specific bin widths: α-terpineol, 0.005 (top) and 0.01 (bottom) μg/g FW; linalool, 0.2 μg/g FW; geraniol, 0.2 (top) and 0.05 (bottom) μg/g FW; and *cis*-linalool oxide, 0.0005 (top) and 0.001 (bottom) μg/g FW. (**A**) Frequency distribution of α-terpineol content in the F1 population. (**B**) Frequency distribution of linalool content in the F1 population. (**C**) Frequency distribution of geraniol content in the F1 population. (**D**) Frequency distribution of *cis*-linalool oxide content in the F1 population.

Phenotypic statistical analysis showed that all four monoterpenoid traits exhibited extensive and continuous variation in the F1 population, with concentration ranges spanning 1–2 orders of magnitude (Figure 2). For α-terpineol, the concentration in the 2019 growing season ranged from 0.0001 to 0.81 μg/g fresh weight (FW); in the 2020 growing season, the concentration ranged from 0.0001 to 0.156 μg/g FW. Linalool, geraniol, and *cis*-linalool oxide showed similar extensive variation patterns, with all traits showing different distributions within the segregating population and exhibiting continuous phenotypic variation. The transgressive segregation phenomenon was widely observed in the F1 population, with some progeny showing significantly higher monoterpenoid concentrations than the high-value parent ‘Muscat Hamburg’, providing excellent germplasm resources for grape-aroma molecular breeding.

### 2.3. Construction of Genetic Maps and QTL Association Analysis of Related Traits

Based on the high-quality phenotypic data of the F1 population, we performed QTL mapping analysis to identify the genetic loci controlling monoterpenoid aroma traits. A high-density integrated genetic map was constructed using specific-locus amplified fragment sequencing (SLAF-seq) technology, which contained 7872 high-quality SNP markers distributed across 19 linkage groups of the grape genome, with a total map length of 2131.69 cM and an average marker interval of 0.29 cM, providing a solid foundation for high-resolution QTL mapping [33]. QTL analysis was performed using composite interval mapping method, with the logarithm of odds (LOD) significance threshold set to 3.0 based on 1000 permutation tests at 95% confidence interval.

**Figure 3:**
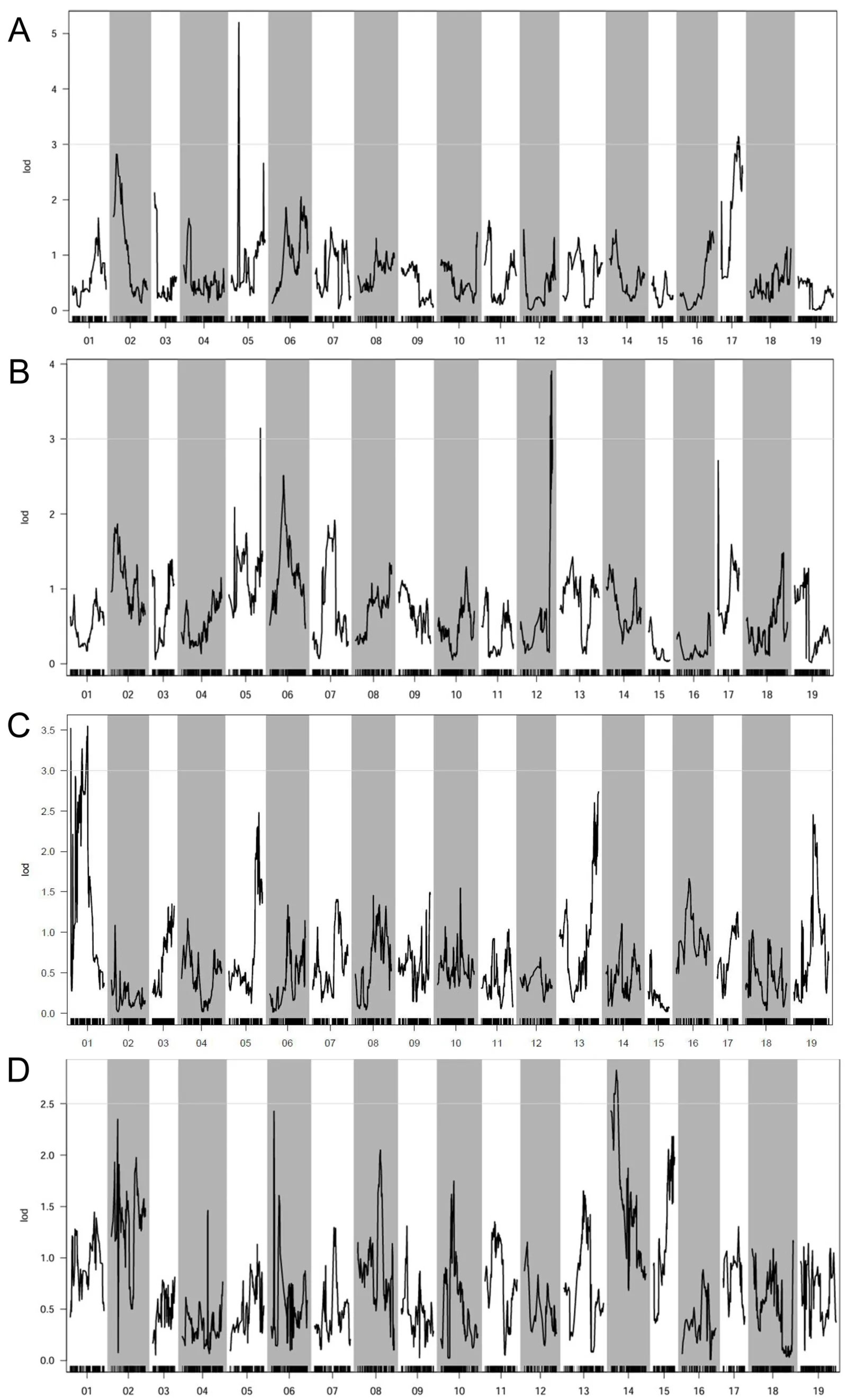
Representative QTL association analysis results for monoterpenoid aroma traits in grape. Manhattan plots show the association signals between single-nucleotide polymorphisms (SNPs) and target aroma traits across the grape genome. The horizontal axis represents the physical position of SNPs on 19 grape chromosomes, the vertical axis represents the negative logarithm of the association *p*-value (−log10(*p*)), and the horizontal dashed line indicates the genome-wide significance threshold. (**A**) QTL association analysis results for α-terpineol content trait in the 2019 growing season (threshold = 3). (**B**) QTL association analysis results for linalool content trait in the 2019 growing season (threshold = 3). (**C**) QTL association analysis results for geraniol content trait in the 2019 growing season (threshold = 3). (**D**) QTL association analysis results for *cis*-linalool oxide content trait in the 2020 growing season (threshold = 2.5).

For the α-terpineol trait, a stable major QTL, which showed a peak LOD of 12.35, was consistently detected on LG5 in both the 2019 and 2020 growing seasons. For the linalool trait, a major QTL was also identified on LG5, overlapping with the α-terpineol QTL interval, with a peak LOD value of 10.82. For the geraniol trait, two significant QTLs were detected on LG1 and LG5, with the LG5 QTL also overlapping with the above-mentioned major interval, indicating that this genomic region on LG5 has a pleiotropic regulatory effect on multiple monoterpenoid aroma traits. For the *cis*-linalool oxide trait, a major stable QTL was detected on LG14 in 2020 growing season, with a peak LOD value of 9.76 (Figure 3 and Figure S2). Given that the LG5 interval was repeatedly detected for α-terpineol and linalool traits across both years and exhibited the highest LOD values, we prioritized this interval for candidate gene screening. The LG14 QTL for *cis*-linalool oxide was also considered for candidate gene analysis due to its stability and high LOD value. Furthermore, the LG01 QTL for geraniol was subjected to candidate gene analysis.

### 2.4. Expression Validation of Candidate Genes Associated with Aroma Traits

Based on the QTL mapping results and grape genome functional annotation, we screened nine candidate genes tightly linked to the major QTL intervals for further expression validation. These genes included four candidate genes for the α-terpineol trait (VIT_05s0020g00760, VIT_05s0020g01240, VIT_05s0020g02030, and VIT_05s0020g02310), one candidate gene for the linalool trait (VIT_05s0020g02130), two candidate genes for the geraniol trait (VIT_01s0011g05960 and VIT_01s0011g05670), and two candidate genes for the *cis*-linalool oxide trait (VIT_14s0036g00050 and VIT_14s0036g00700).

**Figure 4:**
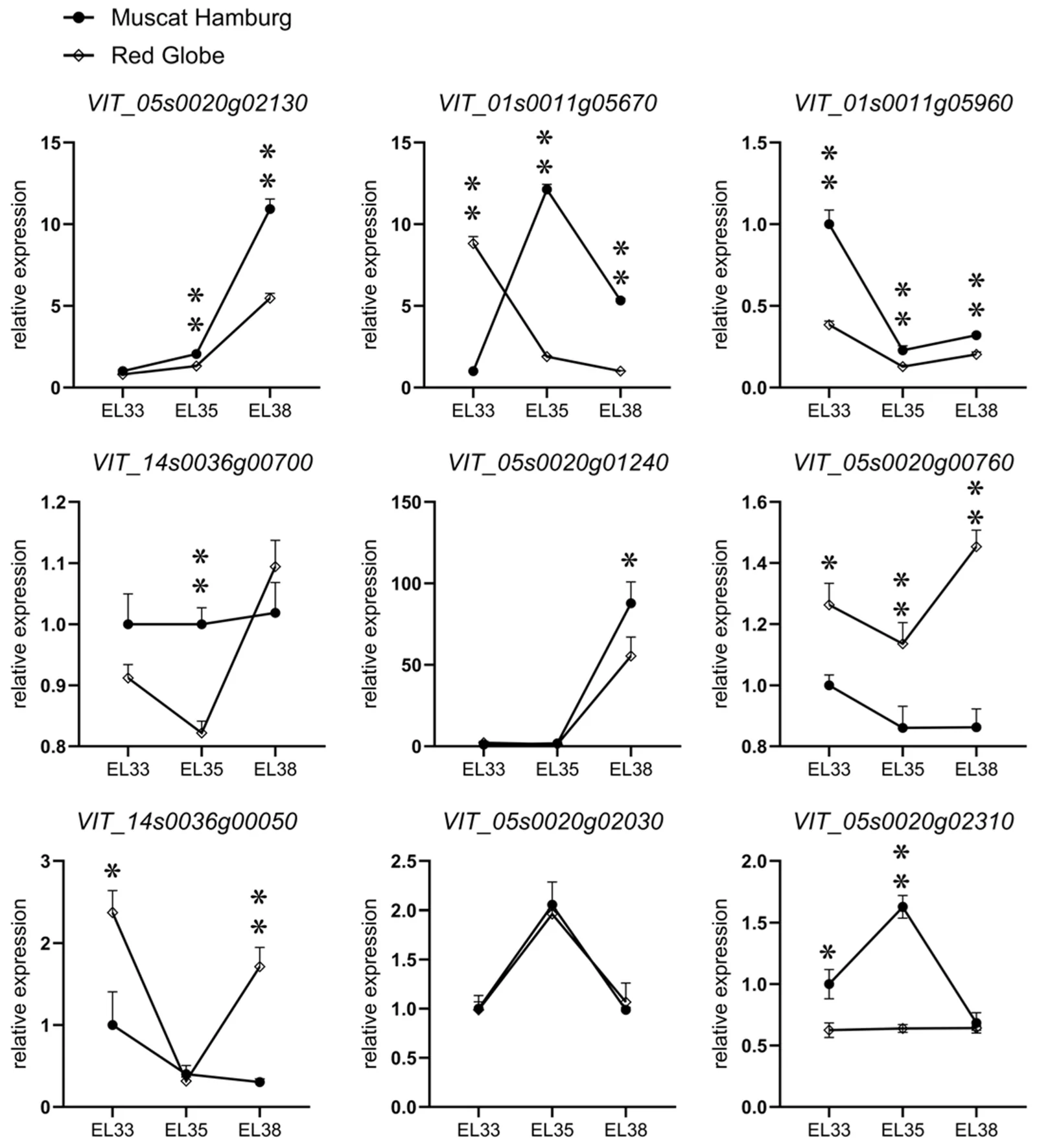
Quantitative real-time PCR validation of candidate genes associated with terpenoid aroma traits identified by QTL mapping. Nine candidate genes linked to terpenoid aroma traits were identified via genetic map construction and QTL association analysis, and their relative expression levels were quantified in ‘Muscat Hamburg’ and ‘Red Globe’ berries across three developmental stages. The error bars represent the standard error (SE) of three biological replicates. Asterisks indicate significant differences as determined by Student’s *t*-test: * *P* < 0.05; ** *P* < 0.01. Candidate genes for α-terpineol trait: *VIT_05s0020g00760*, *VIT_05s0020g01240*, *VIT_05s0020g02030*, and *VIT_05s0020g02310*. Candidate gene for linalool trait: VIT_05s0020g02130. Candidate genes for geraniol trait: *VIT_01s0011g05960* and *VIT_01s0011g05670*. Candidate genes for *cis*-linalool oxide trait: *VIT_14s0036g00050* and *VIT_14s0036g00700*.

We performed quantitative real-time PCR (qRT-PCR) to analyze the relative expression levels of these candidate genes in the berries of ‘Muscat Hamburg’ and ‘Red Globe’ across three key developmental stages (EL33, EL35, and EL38) (Figure 4). The results showed that the expression levels of VIT_05s0020g02130 (annotated as *VvDXS*) and VIT_05s0020g01240 (annotated as *VvGGPPS1*) were significantly higher in ‘Muscat Hamburg’ than in ‘Red Globe’, with the expression difference reaching the maximum at the EL38 stage, which was highly consistent with the accumulation pattern of monoterpenoid aroma components in the two cultivars. The other seven candidate genes showed no significant expression difference between the two cultivars, or their expression patterns were not correlated with the aroma component accumulation trend, and thus they were excluded from subsequent functional validation. These results confirmed that *VvDXS* and *VvGGPPS1* are the most promising candidate genes regulating the aroma formation in ‘Muscat Hamburg’ grape.

### 2.5. Overexpression of VvDXS and VvGGPPS1 Enhanced Berry Aroma in Tomato

**Figure 5:**
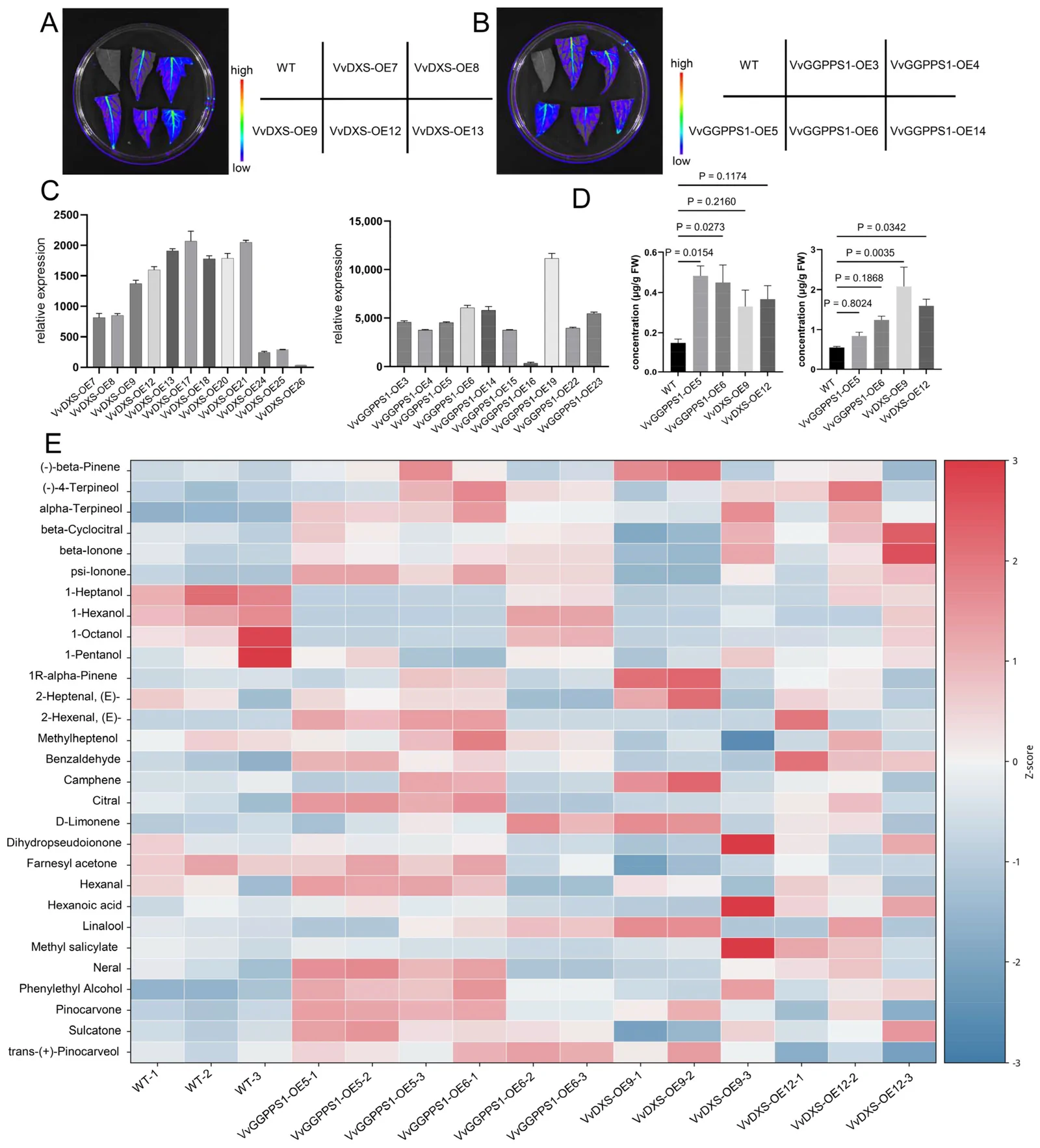
Overexpression of grape *VvDXS* and *VvGGPPS1* genes enhances terpenoid aroma accumulation in tomato fruits. (**A**,**B**) Fluorescence identification of VvDXS-overexpressing (VvDXS-OE) and VvGGPPS1-overexpressing (VvGGPPS1-OE) tomato transgenic lines based on the GFP fluorescent tag. The fluorescence intensity gradient is shown on the right. (**C**) qRT-PCR quantification of *VvDXS* and *VvGGPPS1* relative expression levels in transgenic tomato lines. (**D**) α-terpineol content (left panel) and linalool content (right panel) in mature fruits of WT, VvGGPPS1-OE, and VvDXS-OE tomato lines. Concentrations are expressed as μg/g fresh weight (μg/g FW), error bars represent SE of three biological replicates. (**E**) Cluster heatmap of total volatile aroma component profiles in mature fruits of WT, VvGGPPS1-OE, and VvDXS-OE tomato lines.

To further verify the biological function of *VvDXS* and *VvGGPPS1* in regulating terpenoid aroma biosynthesis, we constructed plant overexpression vectors for the two genes (Figure S3) and performed heterologous overexpression experiments in tomato (*Solanum lycopersicum* L. cv. Micro-Tom), a model plant for fruit aroma research. Transgenic tomato lines were generated via Agrobacterium-mediated transformation, and positive transgenic lines were identified by GFP fluorescence detection (Figure 5A,B) and qRT-PCR quantification of target gene expression levels (Figure 5C). Two independent homozygous overexpression lines with high target-gene expression levels were selected for each gene for subsequent phenotypic and metabolic analysis.

GC-MS quantification of mature tomato fruits showed that overexpression of VvDXS and VvGGPPS1 significantly enhanced the accumulation of terpenoid aroma components in tomato fruits (Figure 5D,E). The α-terpineol concentration in VvDXS-overexpressing (VvDXS-OE) lines was slightly higher than that in the WT, while the linalool concentration was significantly higher than that in WT. For VvGGPPS1-overexpressing (VvGGPPS1-OE) lines, the α-terpineol concentration was significantly increased, and the linalool concentration was slightly increased compared with WT, respectively. No significant difference in fruit size, weight, or other agronomic traits was observed between the transgenic lines and WT, indicating that overexpression of the two genes specifically enhances fruit aroma accumulation without affecting normal fruit development (Figure S4).

A total of 309 volatile metabolites were detected across all samples, of which 29 metabolites were commonly detected in all 15 lines, covering the core volatile components associated with grape aroma, including terpenoids, aliphatic alcohols/aldehydes, aromatic compounds, and esters. To intuitively visualize the difference in volatile metabolite profiles among different lines, we performed Z-score normalization on the relative content of the 29 common volatile components and constructed a clustered heatmap (Figure 5E). HCA of the total volatile metabolite profiles showed a clear separation between VvGGPPS1-OE lines and WT, with the transgenic lines showing significant upregulation of multiple terpenoid and fatty acid-derived aroma compounds (Figure 5E). These results confirmed that *VvDXS* and *VvGGPPS1* positively regulate the biosynthesis and accumulation of terpenoid aroma components in ‘Muscat Hamburg’.

## 3. Discussion

The aroma of table grapes and the persistent and complex fragrance in wine are what modern consumers are seeking [1,34]. The Muscat grape, renowned for its distinctive and unique flavor profile, is currently extensively cultivated in major grape-growing regions across the globe [34]. Within this large and diverse grape cultivar family, ‘Moscato Bianco’ and ‘Muscat of Alexandria’ are widely recognized as the putative primary founding ancestors of the entire Muscat grape lineage [35,36,37]. In some varieties, a number of research efforts have been conducted on aroma components and related regulatory genes, such as *VvDXS1* [17,18,22], *VvbHLH9* [38], and *VvIDI* [39]. However, the existing research references are still insufficient compared to modern grape breeding work. This study used a popular table grape variety known as ‘Muscat Hamburg’. The characteristic muscat aroma of aromatic grape cultivars is primarily driven by monoterpenoid volatile compounds, whose genetic regulatory mechanism remains incompletely elucidated, especially the identification of stable QTLs and functional verification of key regulatory genes in segregating populations [22]. Then we constructed an F1 segregating population derived from a cross between the aromatic cultivar ‘Muscat Hamburg’ and the neutral cultivar ‘Red Globe’, and systematically dissected the genetic basis of grape monoterpenoid aroma traits through integrated volatile metabolomics, high-density genetic map construction, QTL mapping, gene expression validation, and functional verification. Our findings provide a solid theoretical foundation and valuable molecular targets for ‘Muscat Hamburg’ aroma-quality improvement via molecular breeding.

### 3.1. Metabolomic Basis and Phenotypic Genetic Characteristics of Grape Aroma Traits

Volatile metabolomic profiling revealed a clear divergence in berry volatile profiles between ‘Muscat Hamburg’ and ‘Red Globe’, with a total of 442 volatile metabolites detected across all berry developmental stages, of which 127 were significantly differentially abundant between the two cultivars at commercial maturity. The upregulated metabolites in ‘Muscat Hamburg’ were predominantly enriched in the monoterpenoid biosynthesis pathway, consistent with previous studies demonstrating that monoterpenoids are the core contributors to the muscat aroma of grape berries [5,6,11]. The key differential metabolites identified in this study, including *cis*-linalool oxide and α-terpineol, have been widely reported as the primary aroma-active compounds in aromatic grape cultivars [2,40,41,42], further validating the reliability of our metabolomic results. Notably, these monoterpenoids exhibited a continuous upregulation trend during berry ripening in ‘Muscat Hamburg’ but remained at low abundance in ‘Red Globe’, indicating that the biosynthesis of these aroma compounds is developmentally regulated during grape berry maturation.

Linalool and geraniol are key contributing monoterpenols responsible for the typical floral and muscat-like sensory attributes of grape berries [2,7,17]. Free linalool and geraniol accumulate at high concentrations above odor thresholds in muscat-aromatic cultivars but remain at trace levels in neutral grape genotypes, serving as important biochemical markers for muscat aroma phenotype [42]. Owing to their central role in shaping grape berry flavor, linalool and geraniol have been prioritized traits for QTL identification to dissect the genetic architecture underlying monoterpenol-derived aroma in grapevine.

Phenotypic genetic analysis of the F1 segregating population showed that the four core monoterpenoid traits (α-terpineol, linalool, geraniol, and *cis*-linalool oxide) all exhibited continuous and positively skewed normal distributions, failing to fit the standard normal distribution. This is a typical genetic characteristic of plant quantitative traits [17,18], indicating that these aroma traits are controlled by multiple genes with major-effect QTLs playing a dominant regulatory role [22]. These monoterpenoid traits have exhibited extensive and continuous variation in the F1 population, making them ideal targets for QTL mapping.

### 3.2. Reliability of High-Density Genetic-Map Construction and QTL Mapping

The high-density integrated genetic map constructed in this study contained 7872 high-quality SNP markers distributed across 19 linkage groups, with a total map length of 2131.69 cM and an average marker interval of 0.29 cM, representing favorable marker densities for grape-aroma QTL studies. This high-density map improved the accuracy and resolution of QTL mapping, providing a robust foundation for the subsequent identification of key regulatory loci.

The major QTL interval on LG5 was repeatedly detected in both growing seasons, exhibiting significant regulatory effects on α-terpineol, linalool, and geraniol. This QTL interval contained the 1-deoxy-D-xylulose-5-phosphate synthase gene *VvDXS*, which has been reported as a key rate-limiting enzyme in the monoterpenoid biosynthesis pathway, consistent with previous studies on grape aroma regulation [17,18,19,22]. This result indicates that this genomic region harbors a pleiotropic regulatory gene with effects on multiple monoterpenoid aroma traits. Notably, this LG5 interval completely overlaps with the major monoterpenoid aroma QTL regions identified in pioneering studies, further validating the reliability of our QTL mapping results. The stable major-effect QTL on LG5 for α-terpineol and linalool observed over two seasons points to a pleiotropic genomic region controlling multiple muscat-related monoterpenoids. This interval overlaps the earlier studies [17,18,19], confirming its conserved role for monoterpenol accumulation. Importantly, the newly identified stable QTL for *cis*-linalool oxide on LG14 offers a valuable novel target for further gene discovery and aroma-oriented molecular breeding. Our QTL findings illustrate that monoterpenoid biosynthesis is governed by both conserved large-effect loci and previously under-explored secondary genomic regions.

### 3.3. Functional Verification and Biological Significance of Core Regulatory Genes

Based on the QTL mapping results and grape-genome functional annotation, we screened nine candidate genes tightly linked to the major QTL intervals for expression validation. qRT-PCR results revealed that the expression levels of *VvDXS* and *VvGGPPS1* were higher in ‘Muscat Hamburg’ than in ‘Red Globe’ across all berry developmental stages, with the expression difference peaking at the commercial maturity stage. This expression pattern was highly consistent with the accumulation trend of monoterpenoid aroma compounds in the two cultivars, identifying these two genes as the core candidate regulators of grape monoterpenoid aroma biosynthesis. The coordinated upregulation of both VvDXS (the first committed enzyme of the MEP pathway) and VvGGPPS1 (a downstream branch-point enzyme) in ‘Muscat Hamburg’ suggests that the aroma phenotype may involve the coordinated regulation of multiple steps in the terpenoid biosynthesis pathway, rather than being controlled only by VvDXS. The remaining seven candidate genes showed no significant expression differences between the two cultivars, or their expression patterns were not consistent with the accumulation of aroma compounds. Therefore, it remains questionable whether they are related to the production of aroma.

Functional verification via heterologous overexpression in tomato demonstrated that overexpression of *VvDXS* and *VvGGPPS1* significantly enhanced the accumulation of terpenoid aroma components in tomato fruits, with no adverse effects on fruit size, weight, or other agronomic traits. VvDXS-OE lines showed significantly higher linalool accumulation, consistent with the well-established role of DXS as a rate-limiting enzyme in the MEP pathway that increases the overall supply of isoprenoid precursors for monoterpenoid biosynthesis. The function of *VvGGPPS1* in the synthesis of monoterpenoids and norisoprenoids has been previously reported [22]. In the present study, VvGGPPS1-OE lines exhibited a broader enhancement of terpenoid compounds, including significant increases in terpineol and multiple other monoterpenoids. The mechanism by which GGPPS overexpression promotes monoterpenoid accumulation may involve increased flux through the MEP pathway, leading to feedback upregulation of upstream intermediates, including GPP; the potential reverse activity or substrate promiscuity of GGPPS generating GPP; or the formation of heterodimeric complexes with GPPS-like small subunits that alter product specificity, as has been reported in other plant species [29,31]. Further biochemical and molecular studies are needed to elucidate the precise mechanism.

In this study, we identified several stable key genetic loci (QTL) that were consistently detected during two consecutive growing seasons in ‘Muscat Hamburg’. Furthermore, their regulatory effects were confirmed through functional validation, thereby ensuring the reliability and practical application value of our research results. We completed a complete research pipeline from volatile metabolomics to QTL mapping, candidate gene validation, and functional verification, systematically dissecting the genetic regulatory mechanism of grape monoterpenoid aroma. And we identified a major QTL for *cis*-linalool oxide on LG14, providing a new research direction for dissecting the genetic regulation of this important aroma compound. Nevertheless, this study focused only on monoterpenoid aroma components, while grape aroma is also determined by other volatile classes such as esters, aldehydes, and alcohols, whose genetic regulatory mechanisms remain to be elucidated.

## 4. Materials and Methods

### 4.1. Plant Materials and Growth Conditions

Hybridization was performed in May 2011 using ‘Muscat Hamburg’ as the female parent and ‘Red Globe’ as the male parent. F_1_ seedlings were transplanted in 2012 to a rain-sheltered multi-span greenhouse at the Kongzhuang experimental station (39°45′12″ N, 119°12′23″ E, altitude 20 m a.s.l.) of the Changli Institute of Fruit Research, Qinhuangdao, China; and the soil type was sandy loam [43]. Vines were trained on a south–north-oriented pergola system at a height of 1.8 m, with plant spacing of 0.8 m × 2.0 m. During winter pruning (generally in late November), 6–8 fruit-bearing shoots were pruned per vine, with only one cluster retained on each shoot. A balanced fertilization protocol was as follows: 100 kg ha^−1^ urea before budbreak; 60 kg ha^−1^ diammonium phosphate and 40 kg ha^−1^ potassium sulfate one week before flowering; 200 kg ha^−1^ diammonium phosphate and 100 kg ha^−1^ potassium humate after fruit set; and 22,500 kg ha^−1^ well-rotted organic fertilizer after winter pruning [33]. Drip irrigation was scheduled according to vine phenological stages. All other vineyard management practices followed local conventional standards.

### 4.2. Sample Collection and Preparation

Three distinct sets of plant materials were used in this study, as detailed below. (1) Parental cultivars for comparative metabolomics: Berry samples of ‘Muscat Hamburg’ and ‘Red Globe’ were collected at five consecutive developmental stages (EL29, EL33, EL35, EL36, and EL38) according to the modified E-L system [44], with three biological replicates per cultivar per stage. These samples were used for non-targeted comparative volatile metabolomics (Section 2.3, Method A). (2) Parental cultivars and F1 segregating population for QTL mapping: Volatile compounds of 264 F1 individuals and parental cultivars were evaluated at commercial maturity (when seeds turned brown) in the 2019 and 2020 growing seasons. For each individual, 100 g of berry flesh was sampled, frozen in liquid nitrogen, and stored at −80 °C. These samples were used for targeted quantification of four key monoterpenoids (Section 2.3, Method B) and subsequent QTL mapping. (3) Parental cultivars for transcript analysis: Berry samples of ‘Muscat Hamburg’ and ‘Red Globe’ were collected at three developmental stages—EL33 (berries still firm and green, 55 days post-anthesis), EL35 (veraison, 70 days post-anthesis), and EL38 (berries harvest-ripe, 90 days post-anthesis)—with three biological replicates per cultivar per stage. These samples were used for qRT-PCR expression analysis (Section 2.5). For all samples, frozen grape berries were ground in liquid nitrogen to a fine powder prior to extraction.

### 4.3. GC-MS Conditions and Volatile Compound Analysis

Two complementary GC-MS methods were employed in this study, corresponding to the two experimental objectives described in Section 2.2.

Method A—Non-targeted comparative metabolomics: After sampling, desorption of the volatile compounds from the fiber coating was carried out in the injection port of the GC apparatus (Model 8890; Agilent, Santa Clara, CA, USA) at 250 °C for 5 min in the splitless mode. The identification and quantification of volatile compounds was carried out using an Agilent Model 8890 GC and a 7000 D mass spectrometer (Agilent, Santa Clara, CA, USA), equipped with a 30 m × 0.25 mm × 0.25 μm DB-5MS (5% phenyl-polymethylsiloxane) capillary column. Helium was used as the carrier gas at a linear velocity of 1.2 mL/min. The injector temperature was kept at 250 °C, and the detector at 280 °C. The oven temperature was programmed from 40 °C (3.5 min), increasing at 10 °C/min to 100 °C, at 7 °C/min to 180 °C, at 25 °C/min to 280 °C, hold for 5 min. Mass spectra were recorded in electron impact (EI) ionization mode at 70 eV. The quadrupole mass detector, ion source, and transfer line temperatures were set, respectively, at 150, 230, and 280 °C. Selected ion monitoring (SIM) mode was used for MS data acquisition to identify and quantify analytes. This method was used to profile 442 volatile metabolites across the five developmental stages of ‘Muscat Hamburg’ and ‘Red Globe’.

Method B—Targeted quantification of key monoterpenoids (parental cultivars and F1 population): For each individual, 5 g of frozen berry powder was mixed with 1 g sodium chloride and 10 μL of 4-methyl-2-pentanol (internal standard) in a 20 mL capped glass vial. Headspace solid-phase microextraction was performed using an AOC-6000 autosampler (Shimadzu, Kyoto, Japan) equipped with a 2 cm DVB/CAR/PDMS 50/30 μm SPME fiber (Supelco, Bellefonte, PA, USA). After incubation at 40 °C for 30 min, the SPME fiber was inserted into the headspace to absorb volatiles for 30 min, followed by desorption at 230 °C for 8 min [45]. Volatile compounds were identified and quantified using a GC-MS system (QP 2010, Shimadzu, Kyoto, Japan) equipped with an HP-INNOWAX capillary column (60 m × 0.25 mm, 0.25 μm, J&W Scientific, Folsom, CA, USA). Separation was performed in splitless mode with an injector temperature of 230 °C. Helium was used as the carrier gas at a flow rate of 1.0 mL/min. The column oven temperature was programmed as follows: initial temperature of 50 °C held for 1 min, increased to 220 °C at a rate of 3 °C/min, and held at 220 °C for 5 min. The mass spectrometer was operated with an ion-source temperature of 230 °C, an ionization voltage of 70 eV, and a scan range of m/z 30–350. Volatile compounds were identified by comparing their mass spectra with those of authentic standards and the literature data; retention indices were calculated using the Automated Mass Spectral Deconvolution and Identification System. Quantification was performed using external standard calibration curves. For compounds without available authentic standards, concentrations were estimated using standard equations established for compounds with the same functional group or similar carbon atom number [43]. This method was used to quantify the four key monoterpenoids—terpineol, linalool, geraniol, and *cis*-linalool oxide—in the parental cultivars and 264 F1 individuals over two growing seasons.

### 4.4. QTL Mapping for Target Traits

To identify QTLs for cis-linalool oxide, α-terpineol, and geraniol contents, a cross-pollinated segregating population consisting of 264 progenies derived from a cross between ‘Muscat Hamburg’ and ‘Red Globe’ was used. Genomic DNA was extracted using the CTAB method, and only DNA samples with an A260/A280 ratio between 1.8 and 2.0 and a concentration > 50 ng/µL, as assessed by agarose gel electrophoresis and NanoDrop spectrophotometry(Thermo Fisher Scientific, Waltham, MA, USA), were used for subsequent library construction [33]. High-density molecular markers were developed based on whole-genome resequencing of the two parents and specific-locus amplified fragment sequencing (SLAF) of the 264 progenies. A high-density integrated genetic map spanning 2131.69 centimorgans (cM) was constructed using 7872 SNP markers distributed across 19 linkage groups [33]. QTL analysis was performed using composite interval mapping in MapQTL 6.0. The logarithm of odds (LOD) threshold for determining the statistical significance of each QTL was set by 1000 permutation tests. Based on these permutations, the LOD threshold for the three traits was set to 3.0 at a 95% confidence interval. Genes within the QTL intervals were identified by marker mapping, with functional annotation and analysis performed using NCBI databases (https://www.ncbi.nlm.nih.gov/).

### 4.5. Transcription Analysis of Candidate Genes in ‘Muscat Hamburg’

At the three developmental stages corresponding to EL33 (berries still firm and green, 55 days post-anthesis), EL35 (véraison, 70 days post-anthesis), and EL38 (berries harvest-ripe, 90 days post-anthesis) [44], randomly selected berries were used for further experiments. The relevant qRT-PCR quantitative experiments were completed using reagent kits from Vazyme Biotechnology Co., Ltd. (Nanjing, China). The fluorescence quantitative detection was carried out using the ChamQ Universal SYBR qPCR Master Mix (Q711-02) reagent kit (Vazyme, Nanjing, China). The special RT-qPCR primers used are listed in Table S1.

### 4.6. Overexpression of VvDXS and VvGGPPS1 in Tomato

The full-ORF VvDXS was amplified using the forward primer (5′–CGGGGGACTCTTGACCAATGGCTCTCTGTACGCTCTC–3′) and the reverse primer (5′–CGTCATCCTTGTAGTCGGTTAACATGATCTCCAGGGCCTC–3′); the full-ORF VvGGPPS1 was amplified using the forward primer (5′–CGGGGGACTCTTGACCAATGCTTGAGAAGGCTAAGTCAG–3′) and the reverse primer (5′–CGTCATCCTTGTAGTCGGTGTTTTGTCTGTAAGTAGCGAAGTTC–3′). Using the above primer pairs, the target genes were amplified from ‘Muscat Hamburg’ grape cDNA by PCR.

*VvDXS* and *VvGGPPS1* were inserted into the pCAMBIA1301 vector and then mobilized into *Agrobacterium tumefaciens* strain GV3101 for tomato (*Solanum lycopersicum* L. cv. Micro-Tom) transformation. Tomato transformation was carried out via the leaf-disc method, following standard methods [46]. The leaves at the top of the strain (T0) were analyzed to assess the effectiveness of the GFP vector using a plant living molecular marker imaging system (PlantView100, BLT photon technology, Guangzhou, China). Plants were cultivated under greenhouse conditions with a photoperiod of 18 h light at 25 °C and 6 h dark at 18 °C, and a relative humidity of 65%. Two independent transgenic lines (T2), along with wild-type (WT) plants, were used in the experiments. Mature fruits (red-ripe stage) from each line were harvested for phenotypic evaluation and GC-MS analysis, with three biological replicates per line.

### 4.7. Statistical Analysis

Data are presented herein as the mean ± standard error (SE). Differences between two groups were assessed using Student’s *t*-test, while comparisons among multiple groups were conducted by one-way analysis of variance (ANOVA) followed by Duncan’s multiple range test. *p* < 0.05 was considered significant. All statistical analyses were performed using SPSS 22.0 (IBM, Armonk, NY, USA). Figures were generated with GraphPad Prism 9.0 and Adobe Illustrator CS3 13.0.

## 5. Conclusions

In conclusion, this study systematically dissected the metabolic basis and genetic regulatory mechanism of ‘Muscat Hamburg’ monoterpenoid aroma traits. Volatile metabolomic profiling analysis identified that the upregulated metabolites in ‘Muscat Hamburg’ were mainly enriched in the monoterpenoid biosynthesis pathway. Based on these key monoterpenoid aroma phenotypic traits, we identified stable major QTLs for monoterpenoid aroma traits, and validated two core functional genes, *VvDXS* and *VvGGPPS1*, which positively regulate the biosynthesis and accumulation of monoterpenoid aroma compounds in ‘Muscat Hamburg’ berries. Our findings provide valuable theoretical insights and molecular targets for the genetic improvement of grape-aroma quality.

## Data Availability

The raw data supporting the conclusions of this article will be made available by the authors on request.

## Supplementary Materials

The following supporting information can be downloaded at https://www.mdpi.com/article/10.3390/plants15172708/s1, Figure S1. Cluster heatmap analysis of volatile metabolite profiles across five consecutive developmental stages of grape berries; Figure S2. Representative QTL association analysis results for monoterpenoid aroma traits in grape; Figure S3. Schematic maps of the plant overexpression vectors constructed in this study; Figure S4. Statistical analysis of fruit weight, longitudinal diameter, and transverse diameter in transgenic tomato lines overexpressing grape VvDXS and VvGGPPS1; Table S1. Primers used for qRT-PCR analysis of candidate genes associated with terpenoid aroma traits in grape.

## References

[1] McGill C.R., Keast D.R., Painter J.E., Romano C.S., Wightman J.D. (2013). Improved Diet Quality and Increased Nutrient Intakes Associated with Grape Product Consumption by U.S. Children and Adults: National Health and Nutrition Examination Survey 2003 to 2008. J. Food Sci..

[2] Tina I., Danièle W.-R., Nicolas N. (2016). Meta-Analysis of the Core Aroma Components of Grape and Wine Aroma. Front. Plant Sci..

[3] Emanuelli F., Battilana J., Costantini L., Le Cunff L., Boursiquot J.M., This P., Grando M.S. (2010). A candidate gene association study on muscat flavor in grapevine (*Vitis vinifera* L.). BMC Plant Biol..

[4] Robinson A.L., Boss P.K., Solomon P.S., Trengove R.D., Heymann H., Ebeler S.E. (2014). Origins of grape and wine aroma. Part 1. Chemical components and viticultural impacts. Am. J. Enol. Vitic..

[5] Mateo J.J., Jiménez M. (2000). Monoterpenes in grape juice and wines. J. Chromatogr. A.

[6] Wu G., Xin Y.C., Ren R.H., Chen H.W., Yang B.W., Ge M.S., Xie S. (2025). Comprehensive aroma profiles and the underlying molecular mechanisms in six grape varieties with different flavors. Front. Plant Sci..

[7] Li Y., He L., Song Y., Zhang P., Chen D., Guan L., Liu S. (2023). Comprehensive study of volatile compounds and transcriptome data providing genes for grape aroma. BMC Plant Biol..

[8] Panighel A., Vedova A.D., Rosso M.D., Gardiman M., Flamini R. (2010). A solid-phase microextraction gas chromatography/ion trap tandem mass spectrometry method for simultaneous determination of ‘foxy smelling compounds’ and 3-alkyl-2-methoxypyrazines in grape juice. Rapid Commun. Mass Spectrom..

[9] Yang C., Wang Y., Liang Z., Fan P., Wu B., Yang L., Wang Y., Li S. (2009). Volatiles of grape berries evaluated at the germplasm level by headspace-SPME with GC–MS. Food Chem..

[10] Li Z., Howell K., Fang Z., Zhang P. (2019). Sesquiterpenes in grapes and wines: Occurrence, biosynthesis, functionality, and influence of winemaking processes. Compr. Rev. Food Sci. Food Saf..

[11] Mele M.A., Kang H.M., Lee Y.T., Islam M.Z. (2021). Grape terpenoids: Flavor importance, genetic regulation, and future potential. Crit. Rev. Food Sci. Nutr..

[12] Zhou X., Liu S., Gao W., Hu B., Zhu B., Sun L. (2022). Monoterpenoids Evolution and MEP Pathway Gene Expression Profiles in Seven Table Grape Varieties. Plants.

[13] Wang C., Chen Q., Fan D., Li J., Wang G., Zhang P. (2016). Structural Analyses of Short-Chain Prenyltransferases Identify an Evolutionarily Conserved GFPPS Clade in Brassicaceae Plants. Mol. Plant.

[14] Han X., Chen C.C., Kuo C.J., Huang C.H., Zheng Y., Ko T.P., Zhu Z., Feng X., Wang K., Oldfield E. (2015). Crystal structures of ligand-bound octaprenyl pyrophosphate synthase from Escherichia coli reveal the catalytic and chain-length determining mechanisms. Proteins.

[15] Wang Y.C., Wei Y., Li X.Y., Zhang H.M., Meng X., Duan C.Q., Pan Q.H. (2024). Ethylene-responsive VviERF003 modulates glycosylated monoterpenoid synthesis by upregulating VviGT14 in grapes. Hortic. Res..

[16] Doligez A., Adam-Blondon A.F., Cipriani G., Laucou V., Merdinoglu D., Meredith C.P., Riaz S., Roux C., This P., Di Gaspero G. (2006). An integrated SSR map of grapevine based on five mapping populations. Theor. Appl. Genet..

[17] Battilana J., Costantini L., Emanuelli F., Sevini F., Segala C., Moser S., Velasco R., Versini G., Grando M.S. (2009). The 1-deoxy-d-xylulose 5-phosphate synthase gene co-localizes with a major QTL affecting monoterpene content in grapevine. Theor. Appl. Genet..

[18] Duchene E., Butterlin G., Claudel P., Dumas V., Jaegli N., Merdinoglu D. (2009). A grapevine (*Vitis vinifera* L.) deoxy-d-xylulose synthase gene colocates with a major quantitative trait loci for terpenol content. Theor. Appl. Genet..

[19] Battilana J., Emanuelli F., Gambino G., Gribaudo I., Gasperi F., Boss P.K., Grando M.S. (2011). Functional effect of grapevine 1-deoxy-D-xylulose 5-phosphate synthase substitution K284N on Muscat flavour formation. J. Exp. Bot..

[20] Bai C., Capell T., Berman J., Medina V., Sandmann G., Christou P., Zhu C. (2016). Bottlenecks in carotenoid biosynthesis and accumulation in rice endosperm are influenced by the precursor-product balance. Plant Biotechnol. J..

[21] Jiang M., Stephanopoulos G., Pfeifer B.A. (2012). Toward biosynthetic design and implementation of Escherichia coli-derived paclitaxel and other heterologous polyisoprene compounds. Appl. Environ. Microbiol..

[22] Zhang H.M., Lyu X.J., Sun Z.Y., Sun Q., Wang Y.C., Sun L., Xu H.Y., He L., Duan C.Q., Pan Q.H. (2025). GWAS identifies a molecular marker cluster associated with monoterpenoids in grapes. Hortic. Res..

[23] Hemmi H., Noike M., Nakayama T., Nishino T. (2003). An alternative mechanism of product chain-length determination in type III geranylgeranyl diphosphate synthase. Eur. J. Biochem..

[24] Bouvier F., Rahier A., Camara B. (2005). Biogenesis, molecular regulation and function of plant isoprenoids. Prog. Lipid Res..

[25] Chang T.H., Guo R.T., Ko T.P., Wang H.J., Liang P.H. (2006). Crystal Structure of Type-III Geranylgeranyl Pyrophosphate Synthase from Saccharomyces cerevisiae and the Mechanism of Product Chain Length Determination. J. Biol. Chem..

[26] Kloer D.P., Welsch R., Beyer P., Schulz G.E. (2006). Structure and reaction geometry of geranylgeranyl diphosphate synthase from *Sinapis alba*. Biochemistry.

[27] Artz J.D., Wernimont A.K., Dunford J.E., Schapira M., Dong A., Zhao Y., Lew J., Russell R.G., Ebetino F.H., Oppermann U. (2011). Molecular characterization of a novel geranylgeranyl pyrophosphate synthase from Plasmodium parasites. J. Biol. Chem..

[28] Wallrapp F.H., Pan J.J., Ramamoorthy G., Almonacid D.E., Hillerich B.S., Seidel R., Patskovsky Y., Babbitt P.C., Almo S.C., Jacobson M.P. (2013). Prediction of function for the polyprenyl transferase subgroup in the isoprenoid synthase superfamily. Proc. Natl. Acad. Sci. USA.

[29] Zhou F., Wang C.Y., Gutensohn M., Jiang L., Zhang P., Zhang D.B., Dudareva N., Lu S. (2017). A recruiting protein of geranylgeranyl diphosphate synthase controls metabolic flux toward chlorophyll biosynthesis in rice. Proc. Natl. Acad. Sci. USA.

[30] Kavanagh K.L., Dunford J.E., Bunkoczi G., Russell R.G.G., Oppermann U. (2006). The crystal structure of human geranylgeranyl pyrophosphate synthase reveals a novel hexameric arrangement and inhibitory product binding. J. Biol. Chem..

[31] Chang T.H., Hsieh F.L., Ko T.P., Teng K.H., Liang P.H., Wang A.H. (2010). Structure of a heterotetrameric geranyl pyrophosphate synthase from mint (Mentha piperita) reveals intersubunit regulation. Plant Cell.

[32] Leng X., Cong J., Cheng L., Wan H., Liu Y., Yuan Y., Fang J. (2023). Identification of key gene networks controlling monoterpene biosynthesis during grape ripening by integrating transcriptome and metabolite profiling. Hortic. Plant J..

[33] Jia N., Yin Y., Liu C., Han B., Sun Y., Han S., Wang X., Shi P., Zeng Q., Li M. (2026). QTL mapping for berry firmness and functional characterization of a prioritized candidate gene VvNAC83 in grape. Theor. Appl. Genet..

[34] Alem H., Rigou P., Schneider R., Ojeda H., Torregrosa L. (2019). Impact of agronomic practices on grape aroma composition: A review. J. Sci. Food Agric..

[35] Crespan M., Milani N. (2001). The Muscats: A molecular analysis of synonyms, homonyms and genetic relationships within a large family of grapevine cultivars. Vitis.

[36] Myles S., Boyko A.R., Owens C.L., Brown P.J., Grassi F., Aradhya M.K., Prins B., Reynolds A., Chia J.M., Ware D. (2011). Genetic structure and domestication history of the grape. Proc. Natl. Acad. Sci. USA.

[37] Bacilieri R., Lacombe T., Le Cunff L., Di Vecchi-Staraz M., Laucou V., Genna B., Péros J.P., This P., Boursiquot J.M. (2013). Genetic structure in cultivated grapevines is linked to geography and human selection. BMC Plant Biol..

[38] Sun Q., Liu Z., Wang X., Yan A., Ren J., Wang H., Sun L. (2025). Whole-genome identification and functional analysis of grape bHLH transcription factors: New insights into the regulation of monoterpene biosynthesis. Front. Plant Sci..

[39] Huang L., Zhu Y., Wang M., Xun Z., Ma X., Zhao Q. (2024). Integrative Analysis of Transcriptome and Metabolome Reveals the Regulatory Network Governing Aroma Formation in Grape. Horticulturae.

[40] Liu J., Zhu X.L., Ullah N., Tao Y.S. (2017). Aroma Glycosides in Grapes and Wine. J. Food Sci..

[41] Song M., Fuentes C., Loos A., Tomasino E. (2018). Free Monoterpene Isomer Profiles of *Vitis vinifera* L. cv. White Wines. Foods.

[42] Moriyama K., Kono A., Matsuzaki R., Azuma A., Onoue N., Sekozawa Y., Sato A., Sugaya S. (2024). Diversity of flavour characteristics of table grapes and their contributing volatile compounds analysed by the solvent-assisted flavour evaporation method. Hortic. Res..

[43] Wang X., Liu C., Jia N., Yin Y., Han B., Sun Y., Han S., Guo Y., Li M. (2025). Rootstock-mediated effects on vine performance and quality composition of ‘Miguang’ under protected cultivation in northern China. Eur. J. Hortic. Sci..

[44] Coombe B.G. (2010). Growth Stages of the Grapevine: Adoption of a system for identifying grapevine growth stages. Aust. J. Grape Wine Res..

[45] Wang Y., Chen W.-K., Gao X.-T., He L., Yang X.-H., He F., Duan C.-Q., Wang J. (2019). Rootstock-Mediated Effects on Cabernet Sauvignon Performance: Vine Growth, Berry Ripening, Flavonoids, and Aromatic Profiles. Int. J. Mol. Sci..

[46] Pagliuso D., Rossi M., Freschi L. (2025). Highly Efficient Agrobacterium-Mediated Transformation of Tomato cv Micro-Tom From Cotyledon Explants. Bio-protocol.

