# Supplementary material for: GC-MS-Based Volatile Metabolomics and Quantitative Trait Locus Mapping of Aroma Components in ‘Muscat Hamburg’ Grape
Source: Plants (Basel). 2026 Sep 3;15(17):2708. doi: 10.3390/plants15172708 (PMC13567957; doi:10.3390/plants15172708)
Supplement: Supplementary file 1 [file plants-15-02708-s001.zip › Supplemental Materials .pdf]

Supplementary Materials for

GC-MS-Based Volatile Metabolomics and Quantitative Trait Locus Mapping of Aroma Components in 'Muscat Hamburg' Grape

Minmin Li [1,2,†], Peng-Tao Shi [2,†], Nan Jia [2], Yong-Gang Yin [2], Yan Sun [2], Bin Han [2], Chang-Jiang Liu [2], Shu-Li Han [2], Ying-Jie Wang [2], Xin-Yu Wang [2,*,‡] and Jian-Rong Feng [1,*]

[1] Xinjiang Production and Construction Corps Key Laboratory of Special Fruits and Vegetables Cultivation Physiology and Germplasm Resources Utilization, College of Agriculture, Shihezi University, Shihezi 832000, China; 15903395217@163.com

[2] Changli Institute of Pomology, Hebei Academy of Agriculture and Forestry Sciences, Changli, Qinhuangdao 066600, China; 2018051164@nwafu.edu.cn (P.-T.S.); nannanjia123@163.com (N.J.); yinyonggang@126.com (Y.-G.Y.); sycoolhi@163.com (Y.S.); hanbin986@163.com (B.H.); liuchj2008@163.com (C.-J.L.); h15633401356@163.com (S.-L.H.); 17733503931@163.com (Y.-J.W.)

* Correspondence: cgswxy123@163.com (X.-Y.W.); fjr_agr@shzu.edu.cn (J.-R.F.)

† These authors contributed equally to this work.

‡ The author responsible for distribution of materials integral to the findings presented in this article in accordance with the policy described in the Instructions for Authors is Xin-Yu Wang.

The PDF file includes:

Figure S1. Cluster heatmap analysis of volatile metabolite profiles across five consecutive developmental stages of grape berries.

Figure S2. Representative QTL association analysis results for monoterpenoid aroma traits in grape.

Figure S3 Schematic maps of the plant overexpression vectors constructed in this study.

Figure S4. Statistical analysis of fruit weight, longitudinal diameter, and transverse diameter in transgenic tomato lines overexpressing grape *VvDXS* and *VvGGPPS1*.

Table S1. Primers used for qRT-PCR analysis of candidate genes associated with terpenoid aroma traits in grape.

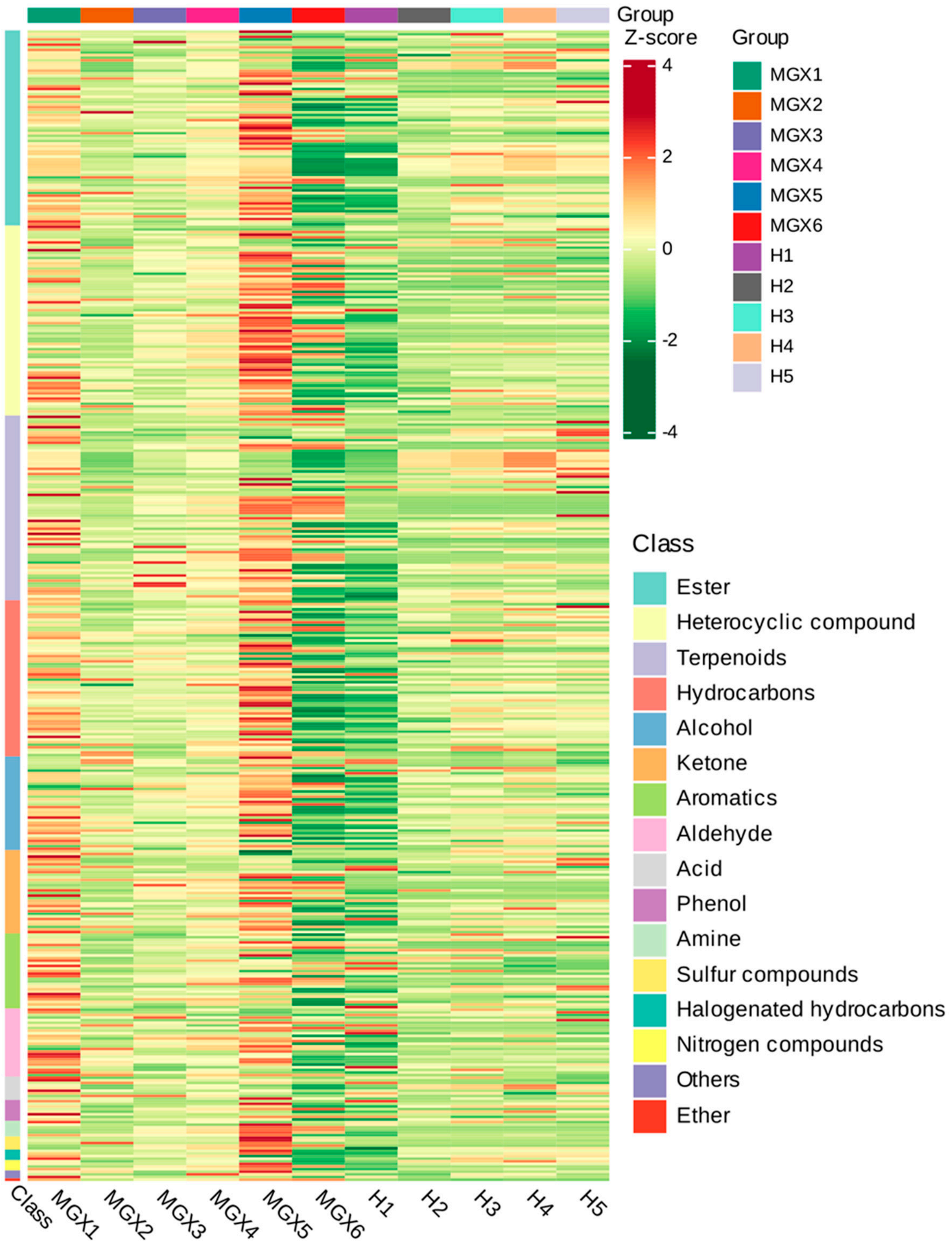

Figure S1. Cluster heatmap analysis of volatile metabolite profiles across five consecutive developmental stages of grape berries.

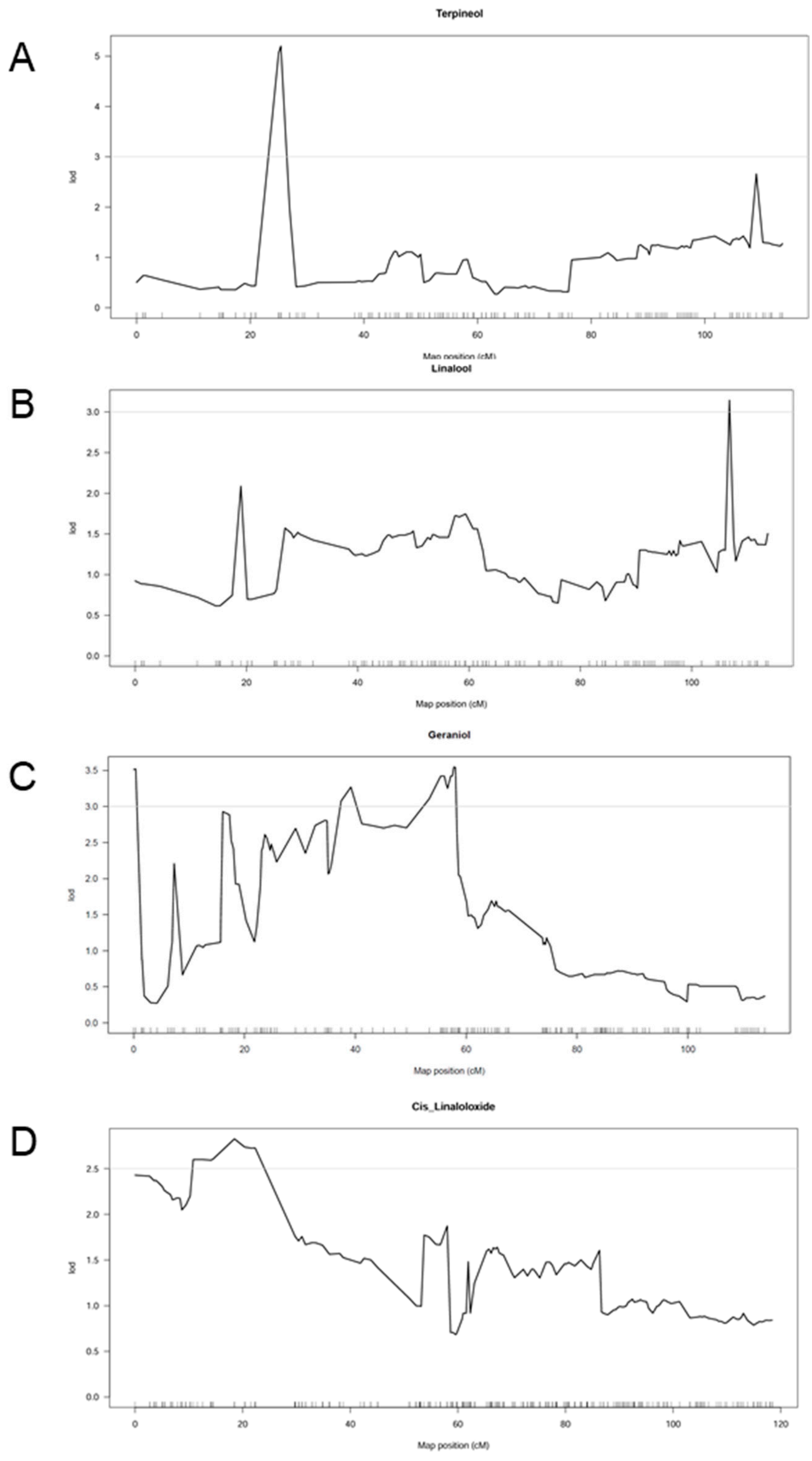

Figure S2. **Representative QTL association analysis results for monoterpenoid aroma traits in grape.** Manhattan plots show the association signals between single nucleotide polymorphisms (SNPs) and target aroma traits across the grape genome. The

horizontal axis represents the physical position of SNPs on target chromosomes, the vertical axis represents the negative logarithm of the association *P*-value (-log10(*P*)), and the horizontal dashed line indicates the genome-wide significance threshold. A, QTL association analysis results for alpha-terpineol content trait in the 2019 growing season (chromosome 05, threshold=3). B, QTL association analysis results for linalool content trait in the 2019 growing season (chromosome 05, threshold=3). C, QTL association analysis results for geraniol content trait in the 2019 growing season (chromosome 01, threshold=3). D, QTL association analysis results for cis-linaloloxide content trait in the 2020 growing season (chromosome 14, threshold=2.5).

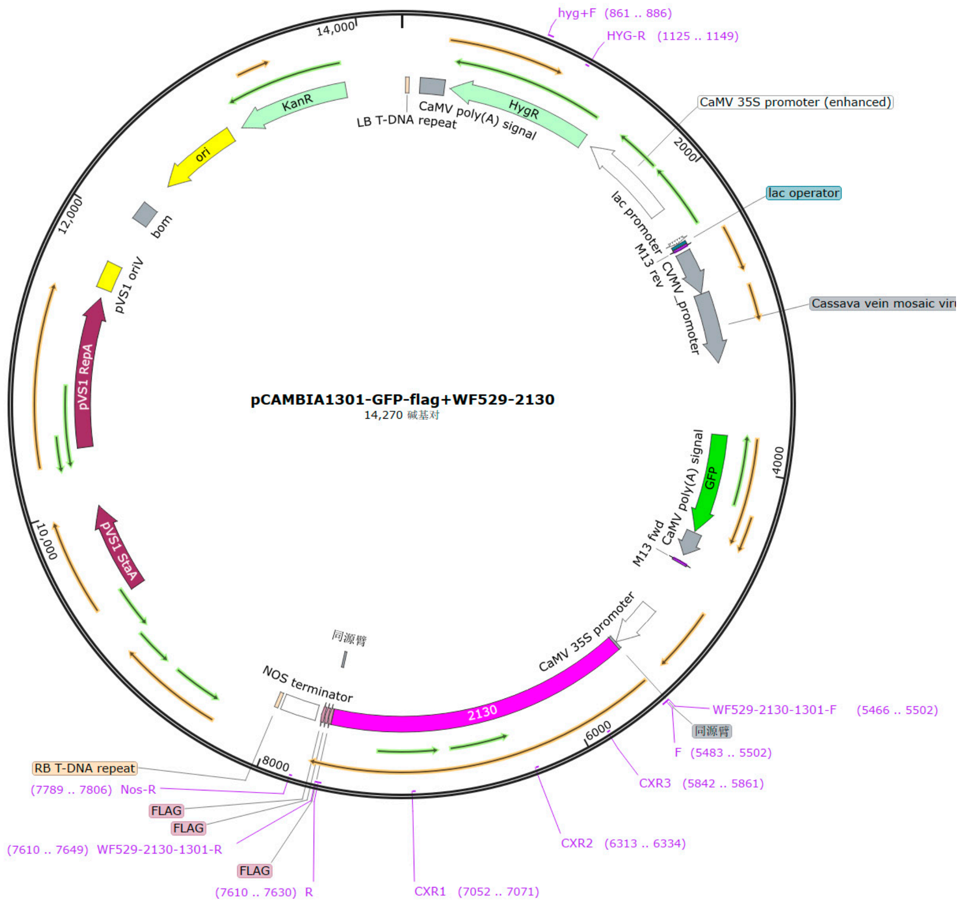

(A) pCAMBIA1301-GFP-flag-VvDXS

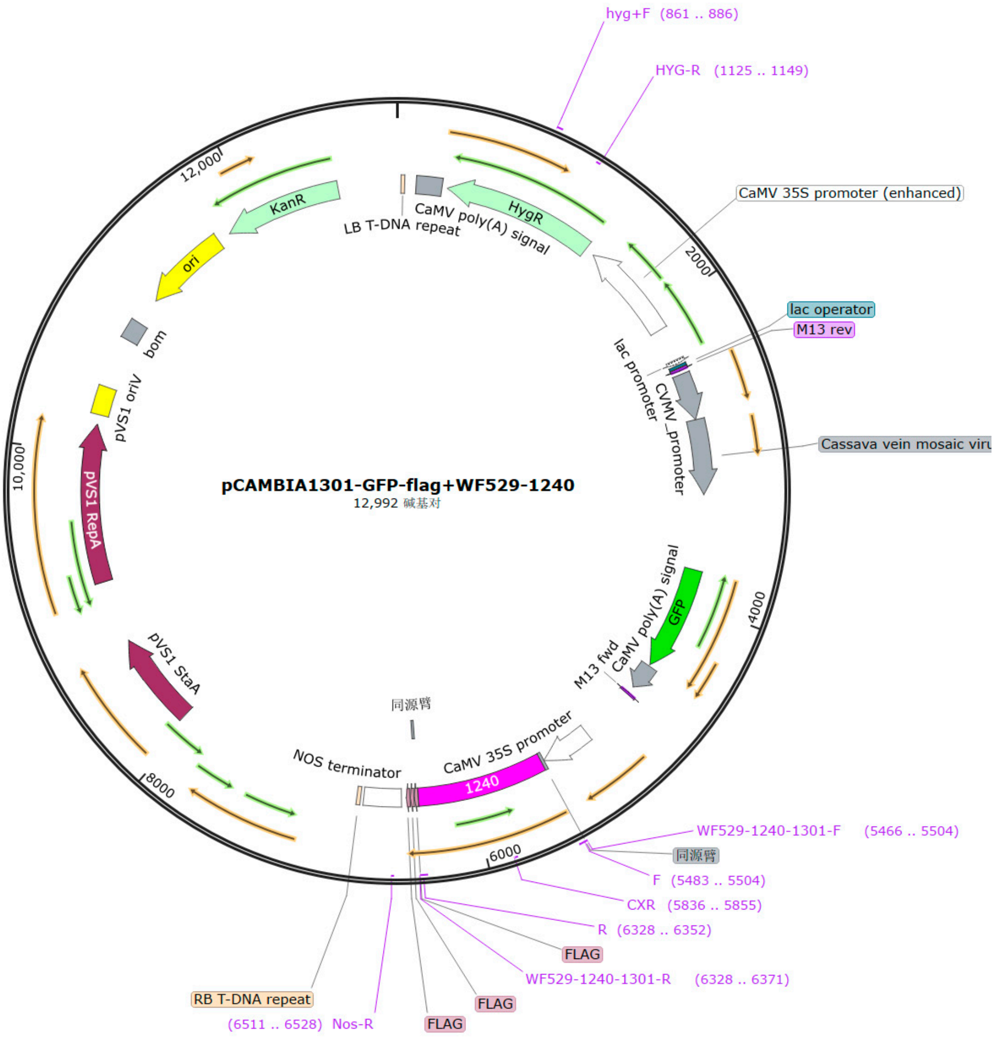

(B) pCAMBIA1301-GFP-flag-VvGGPPS1

Figure S3. Schematic maps of the plant overexpression vectors constructed in this study. A, (A) The pCAMBIA1301-GFP-flag-VvDXS overexpression vector (total length: 14,270 bp), carrying the full-length coding sequence (CDS) of *VvDXS* (VIT_05s0020g02130) under the control of the CaMV 35S promoter. (B) The pCAMBIA1301-GFP-flag-VvGGPPS1 overexpression vector (total length: 12,992 bp), carrying the full-length CDS of *VvGGPPS1* (VIT_05s0020g01240) under the control of the CaMV 35S promoter. Key vector elements include: CaMV 35S promoter (enhanced) for constitutive transgene expression, GFP fluorescent tag for transgenic line screening,

FLAG tag for protein immunodetection, NOS terminator for transcription termination, HygR (hygromycin resistance gene) and KanR (kanamycin resistance gene) for selection, and LB/RB T-DNA border repeats for Agrobacterium-mediated T-DNA transfer. The homologous arm sequences and primer binding sites (CXR1, CXR2, CXR3, WF529-1301-F/R) used for vector construction and validation are also indicated.

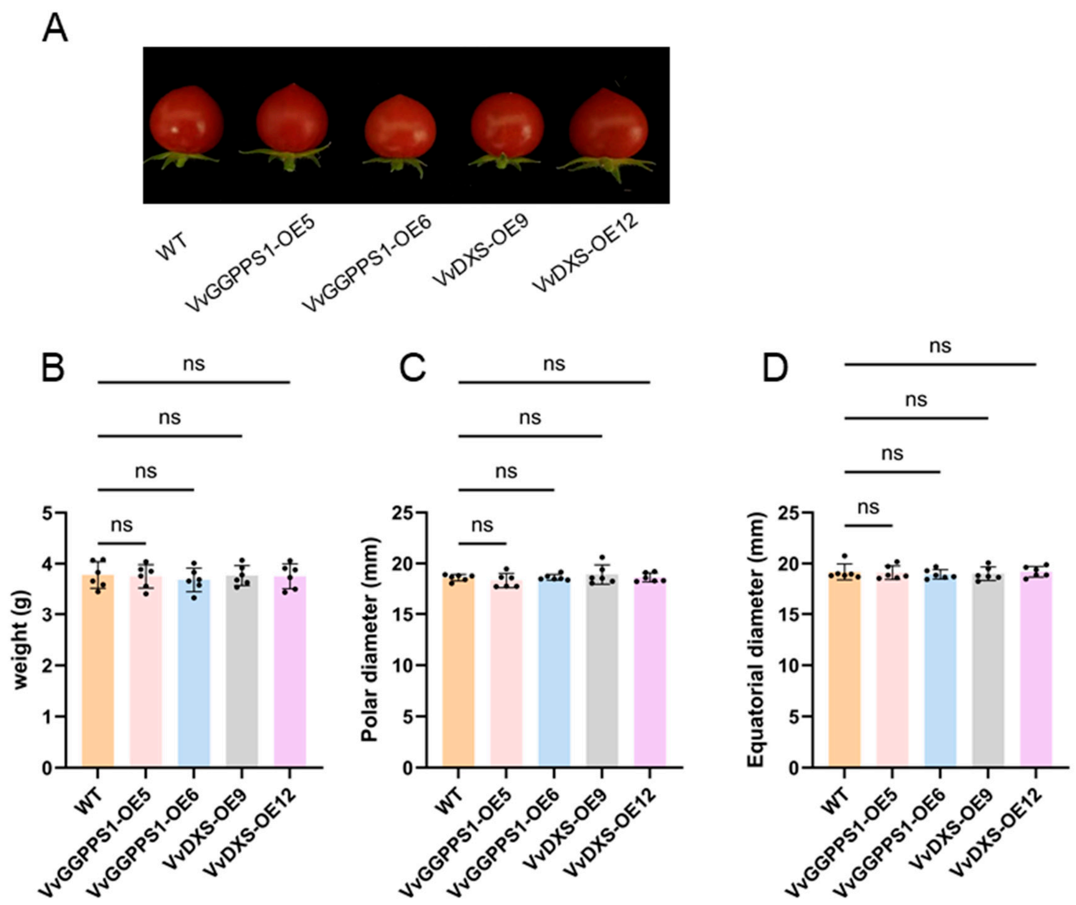

Figure S4. Statistical analysis of fruit weight, longitudinal diameter, and transverse diameter in transgenic tomato lines overexpressing grape *VvDXS* and *VvGGPPS1*. A, Representative images of WT, VvGGPPS1-OE, and VvDXS-OE tomato lines. **(**B) Fruit weight per fruit (g). (C) Longitudinal diameter (mm). (D) Transverse diameter (mm) of mature fruits. Data are presented as means ± standard error (SE). Asterisks indicate statistically significant differences compared with the wild-type (WT) control (one-way ANOVA with Tukey's multiple comparison test; $P < 0.05$, $*P < 0.01$; ns, not significant). OE, overexpression.

Table S1. Primers used for qRT-PCR analysis of candidate genes associated with terpenoid aroma traits in grape.

Gene ID	Gene annotation / trait	Forward primer (5′→3′)	Reverse primer (5′→3′)
VIT_05s0020g00760	Terpineol candidate gene	AATCGCCAGGAGAGCCATTG	GAAGGTCACGGTAAGAGTCCAA
VIT_05s0020g01240	VvGGPPS1 (geranylgeranyl diphosphate synthase 1); Terpineol candidate gene	AGCCATTCCACTGAGAGAAC	CTAGCTCACAAGCGGAGATAC
VIT_05s0020g02030	Terpineol candidate gene	TTGGCAACCTTGTGGTGAGTAT	ACCGGAACTTGAGAATGGACAG
VIT_05s0020g02310	Terpineol candidate gene	AGGAAGGAGTGAGGGCAACA	AGACCAGGCGGCACAGATA
VIT_05s0020g02130	VvDXS (1-deoxy-D-xylulose-5-phosphate synthase); Linalool candidate gene	GTGACCAAGTTCGATCCTGCTA	CCTTGTCCACCTCTGCTTCTG
VIT_01s0011g05960	Geraniol candidate gene	GGTGCAGCGTGACCGATTA	TCAACTGGAGGAGAAGCGAATT
VIT_01s0011g05670	Geraniol candidate gene	ATATGGATGGATGTGGCGGAAG	TGTCGTGGCAGAGTCGTGA
VIT_14s0036g00050	cis-Linalool oxide candidate gene	TCTAGCTGCTGATGCTGATGAG	GCCCTTCTTCACAGTCTCTTCA

VIT_14s0036g00700	cis-Linalool oxide candidate gene	TGACAGGAGAGCAGGTTCTACA	CTGAGCATTAGCCGTCTACGAT
VvActin (reference)	Actin; internal reference gene	CTTGCATCCCTCAGCACCTT	TCCTGTGGACAATGGATGGA
